# Thermally Processable, Transparent, Mechanically Tunable and Robust Bioplastics From Wastepaper

**DOI:** 10.1002/advs.75978

**Published:** 2026-06-09

**Authors:** Zhezhe Zhou, Tao Chu, Boyou Hou, Mark Lynch, Siqi Huo, Min Hong, Jianguo Yang, Paulomi (Polly) Burey, Qiang Gao, Guobo Huang, Pingan Song

**Affiliations:** ^1^ Centre for Future Materials School of Science Engineering and Digital Technologies University of Southern Queensland Springfield Australia; ^2^ School of Electromechanical and Mold Engineering Taizhou Vocational College of Science & Technology Taizhou P. R. China; ^3^ School of Pharmaceutical and Chemical Engineering Taizhou University Jiaojiang P. R. China; ^4^ College of Materials Science and Technology Beijing Forestry University Beijing P. R. China

**Keywords:** biodegradability, bioplastics, thermally processible cellulose, tunable mechanical property, wastepaper

## Abstract

To mitigate the environmental impact of plastic pollution and paper waste, developing bioplastics from wastepaper as alternatives to non‐degradable petroleum‐based plastics is of great importance. However, current wastepaper recycling faces challenges such as complex preparation processes, low efficiency, suboptimal performance, and limited scalability. In this work, we present a facile and scalable direct hot‐press transformation approach to directly convert wastepaper into thermally processable, transparent, and high‐performance bioplastics. This approach involves the cleavage of the cellulose ring structure in wastepaper, followed by hot‐pressing under mild conditions. The resultant bioplastics demonstrate excellent thermal processability and tunable mechanical properties (with tensile strengths ranging from 85.7 to 103.2 MPa) and thus can be converted into rigid containers or flexible packaging bags without the need for adhesives. They also exhibit high optical transparency, good water resistance, repairability and biodegradability. This work provides a streamlined direct transformation strategy to produce thermally processible, transparent and mechanically robust bioplastics, offering a scalable avenue to transform waste papers into bioplastics for sustainable packaging.

## Graphical abstract

Unlike conventional inefficient or low‐value wastepaper recycling, a directional transformation strategy is employed to convert whole wastepaper into intact bioplastics with tunable mechanical properties in our work. These bioplastics can be processed into flexible bags or rigid containers and exhibit high transparency, water resistance, repairability, malleability, and biodegradability.

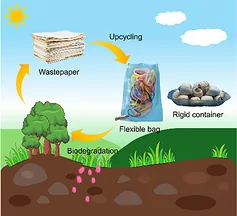

## 1 Introduction

The environmental concerns arising from the increasing accumulation of non‐biodegradable petroleum‐based plastics have driven the development of biodegradable plastics in recent decades. Currently, a variety of bioplastics have been developed from renewable biomass sources, such as starch [1], cellulose [2], and proteins [3]. Typical examples include polylactic acid (PLA) [4], polyhydroxyalkanoates [5], and polybutylene succinate (PBS) [6], which are being commercially produced from corn starch via microbial fermentation. However, their production often involves multiple complicated synthesis processes, i.e., monomer hydrolysis, fermentation, and polymer synthesis, which significantly lead to increased production costs and reduced production efficiency. Also, bioplastics made directly from starch or other biomass still suffer from inherent property limitations, such as poor mechanical strength, low water resistance, and limited thermal stability [7]. Moreover, the large‐scale use of food crops for bioplastic production can compromise food supply and increase production costs [7]. Hence, the production of many existing bioplastics from biomass feedstock has been impeded by their complicated synthesis, unsatisfactory physical properties, and high production costs.

**FIGURE 1:**
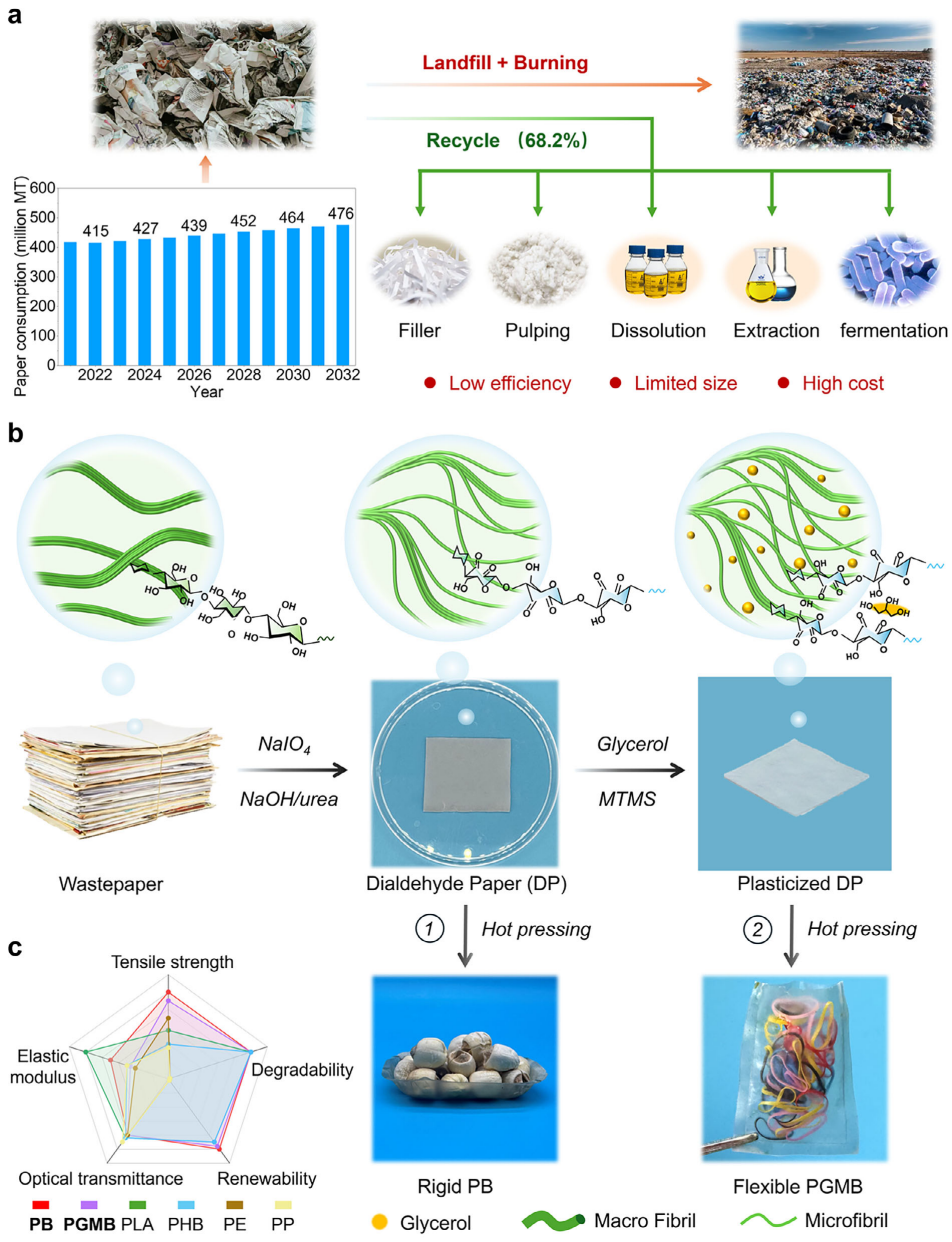
Background and conceptual design of wastepaper‐based bioplastics. (a) Paper consumption from 2021 to 2032 (projected) and management of wastepaper. (b) Schematic illustration of the fabrication routes for rigid PB and flexible PGMB. (c) Comprehensive evaluation of the overall performance of wastepaper‐based bioplastics against representative commercial plastics.

Wastepaper, a lignocellulosic biomass‐derived material, has attracted increasing attention for fabricating bioplastics as one of the most promising raw materials because of its low cost and its main component, cellulose, which offers superior mechanical properties [7]. Recent statistics have estimated that 110 million tons of paper and cardboard waste were managed in the United States in 2019 [8]. Over 400 million tons of paper and paperboard are used worldwide each year, with a projected increase to 476 million tons by 2032 [9]. Hence, the reuse of wastepaper also holds great potential to reduce landfill waste, save trees, lower energy use, and reduce pollution. However, recycled wastepaper is often used merely as a filler in combination with other biomaterials, such as starch, PLA [10], or chitosan [11]. Meanwhile, the preparation of paper‐based bioplastics often involves complex and energy‐intensive steps, including pulping [12], fiber dissolution [13, 14, 15], cellulose extraction [16], and enzymatic hydrolysis or fermentation (Figure 1a) [17]. These processes not only reduce production efficiency and increase costs, thereby hindering scalability, but also frequently involve the release of hazardous chemicals to the environment. Only a few studies design bioplastics by using intact paper, such as soaking or coating with resins [18], cyclic carbonate/amine compounds [19], and polyamines [20]. Nevertheless, these materials normally suffer from low transparency and even non‐transparency, poor durability due to additive leaching, and limited biodegradation conditions. Furthermore, the inherently poor thermal processability of cellulose remains a major challenge, because of strong inter‐ and intra‐molecular hydrogen bonding (H‐bonding) within its dense semicrystalline structure that contributes to a high glass transition temperature (200°C–250°C) [21]. Therefore, it has been highly attractive to develop a facile, efficient, and sustainable strategy to directly convert wastepaper into low‐cost high‐performance transparent bioplastics.

To bridge this research gap, we here demonstrate a direct top‐down conversion of intact wastepaper into thermally processable, mechanically robust, and highly transparent bioplastics (Figure 1b). A frozen NaOH/urea pretreatment is conducted to disrupt the inter‐ and intra‐molecular H‐bonding of cellulose in wastepaper and its hierarchical semicrystalline structure. Subsequently, the treated paper is oxidized to form dialdehyde paper (DP) using NaIO_4_, and this process allows the transformation of the original ring‐rich structure of cellulose into a more linear one, thus enhancing its chain mobility and thermal processability. The resultant DP can be thermally processed into strong and transparent rigid bioplastic film by ready hot‐pressing under a mild temperature (i.e., 110°C). Upon plasticization treatment with glycerol, DP can also be readily thermally processed into flexible transparent bioplastic films with tunable mechanical properties. The mechanically tunable wastepaper‐based bioplastics can be used to produce rigid food containers and flexible packaging bags. This work offers a facile, economically viable and eco‐benign approach to converting wastepaper into high‐performance transparent bioplastic films, which show great application potential as low‐cost biodegradable packaging materials, thus providing a new solution to existing non‐biodegradable petroleum‐based packaging materials.

## 2 Results and Discussion

### 2.1 Fabrication and Chemical Characterization of the Wastepaper‐Based Bioplastics

The NaIO_4_ oxidation disrupts H‐bonding interactions among cellulose chains and cleaves some six‐membered glucose rings in cellulose macromolecules. Consequently, the resulting derivative DP can be thermally molded into rigid, transparent paper‐based bioplastics (PB) by simple hot pressing under mild conditions. These PBs can be further shaped into food containers (Figure 1b). Additionally, plasticizing DP with glycerol and hydrophobic methyltrimethoxysilane (MTMS) produces flexible, transparent, and self‐healing paper/glycerol/MTMS‐based bioplastics (PGMBs) with tunable mechanical properties. A heat‐sealable PGMB packaging bag is also fabricated, suitable for adhesive‐free applications. Both PB and PGMB exhibit excellent mechanical strength, optical transparency, and sustainability (renewability and biodegradability), offering practical and eco‐friendly alternatives to petroleum‐based plastics and outperforming many existing bioplastics (Figure 1c).

**FIGURE 2:**
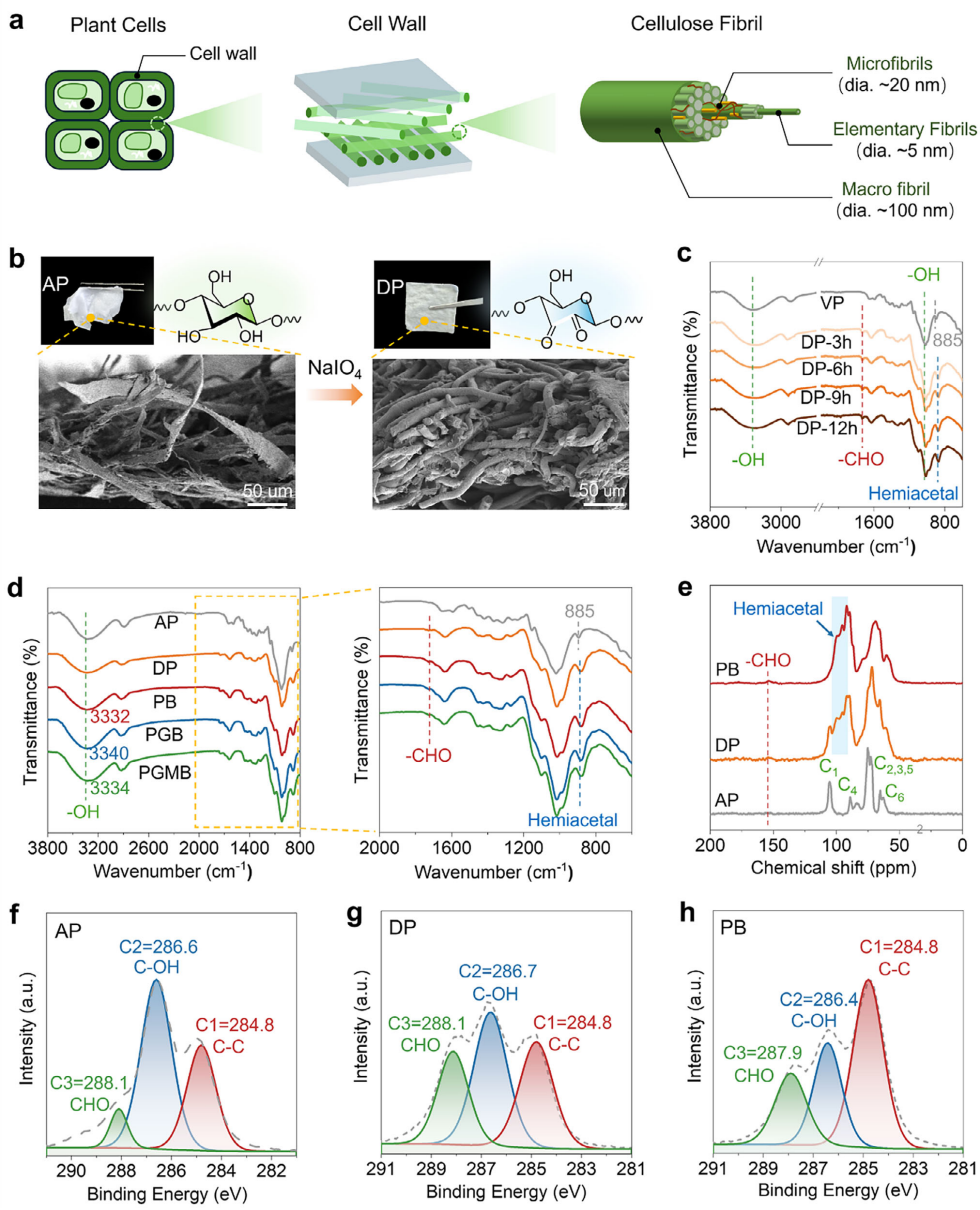
Chemical characterizations of DPs and wastepaper‐based bioplastics. (a) Schematic illustration of the hierarchical cellulose architecture in the plant cell wall. (b) Chemical structures and morphologies of AP and DP. FTIR spectra of (c) DPs and (d) various wastepaper‐based bioplastics. (e) NMR spectra of AP, DP, and PB. XPS spectra of (f) AP, (g) DP, and (h) PB.

It is critical to gain an in‐depth understanding of how the chemical treatments facilitate the thermal processability of wastepaper. The NaOH/urea treatment partially dissolves the dense fibers of virgin wastepaper (VP), generating loose microfibrils on the surface of the alkali‐treated paper (AP) (Figure 2a; Figure S1). This process disrupts the hierarchical fiber structure, increases surface area, and reduces both crystallinity and mass due to partial dissolution of cellulose crystalline regions (Figure S2). Unlike the anisotropic VP, which shows large mechanical differences between the longitudinal and transverse directions, AP exhibits more isotropic strength (Figure S3a,b). The more isotropic mechanical response of AP is likely attributed to a combination of factors, including partial disruption of fiber alignment, increased inter‐fibril interactions, and possible reduction in intrinsic fiber strength induced by alkali treatment (yellow arrows, Figure S3c,d). These microfibrils can regenerate into a robust H‐bonded cellulose network, maintaining structural integrity during oxidation. In contrast, untreated VP collapses or disintegrates upon oxidation due to chain cleavage and insufficient H‐bonding (Figure S4).

To examine the effects of NaIO_4_ oxidation on the chemical structure of alkali‐treated paper, a series of DPs were prepared with varying oxidation times. The C2–C3 bonds in cellulose glucose units are selectively cleaved, replacing the 2,3‐dihydroxyl groups with 2,3‐dialdehyde groups (Figure 2b) [22]. With increasing oxidation time, the size and surface area of DPs decrease while the cross‐sectional width increases (Figure S5), resembling the shrinkage behavior of thermoplastic films [23]—likely due to microstructural changes (Figure S6). Oxidation produces smoother fiber surfaces with larger gaps and pores. This is because NaIO_4_ removes lignin and surface impurities, which weakens inter‐fiber interactions and allows greater fiber mobility. The substitution of ─OH with ─CHO groups also enhance hydrophobicity, causing fiber swelling and increased DP thickness upon drying [24]. Additionally, intramolecular hemiacetal formation between dialdehyde and native cellulose induces non‐linear conformations and structural buckling, leading to macroscopic dimensional changes [25, 26]. These morphological and structural variations clearly reflect the chemical modification of cellulose fibers in wastepaper.

The aldehyde content in DPs increases from 2.01 mmol g^−1^ (DP‐3h) to 8.23 mmol g^−1^ (DP‐12h), showing a clear rise with longer oxidation time (Figure S7). This chemical transformation is further supported by multiple characterizations. As shown in Figure 2c, a new absorption peak at ∼1732 cm^−1^ corresponding to ─CHO groups appear after oxidation [22], with intensity increasing over time, while the peaks at ∼3322 and 1016 cm^−1^ (─O─H and ─C─O─H stretching) decrease markedly. These results confirm the substitution of partial ─OH groups by ─CHO through NaIO_4_ oxidation. The same ─CHO peak is also observed in the FTIR spectra of PB, paper/glycerol‐based bioplastic (PGB), and PGMB (Figure 2d). Likewise, a weak signal at 153.91 ppm, assigned to ─CHO, appears in the NMR spectra of both DP and PB (Figure 2e). XPS analysis of DP and PB further reveals a stronger C═O peak at ∼288.1 eV and a weaker C─O peak at ∼286.6 eV compared to AP, indicating decreased ─OH and increased ─CHO content (Figure 2f–h) [27]. Collectively, these results confirm the successful introduction of aldehyde groups in DPs. Interestingly, side reactions also occur during oxidation. The characteristic peak at 885 cm^−1^ for AP shifts to 871 cm^−1^ in DPs and 881 cm^−1^ in the PB, with increased intensity indicating glucose ring alterations and the formation of hemiacetal and hydrated structures during NaIO_4_ oxidation [28]. The NMR spectrum of AP shows distinct peaks for glucose carbons—C1 (105 ppm), C4 (89 ppm), C2, C3, C5 (73–74 ppm), and C6 (65 ppm)—whereas DP and PB display additional shifts between 85 and 105 ppm, assigned to C atoms in hemiacetal structures (Figure S8) [29, 30]. These results confirm the presence of aldehyde‐related forms such as hydrates, hemiacetals, and hemialdals. PB exhibits more pronounced and diverse peaks in this region than DP, suggesting additional aldehyde‐derived side products form during hot pressing.

Compared with DP, PB shows a stronger IR peak at 3332 cm^−1^ (O─H), indicating enhanced H‐bonding after hot pressing. With glycerol addition, this peak blue‐shifts to 3340 cm^−1^ and further intensifies in PGB, reflecting more free ─OH groups. XPS analysis likewise reveals a stronger C2 peak (C─OH) in PGB than in PB, confirming the introduction of additional ─OH groups from glycerol (Figure S9). No significant structural changes are observed between PGB and PB, suggesting glycerol is physically incorporated rather than chemically bonded. After hydrophobic surface modification, PGMB exhibits a stronger C1 peak (C─C) than PGB, consistent with the abundant C─C bonds from silane groups on the PGMB surface.

### 2.2 Crystallinity and Dynamic Mechanical Analysis

**FIGURE 3:**
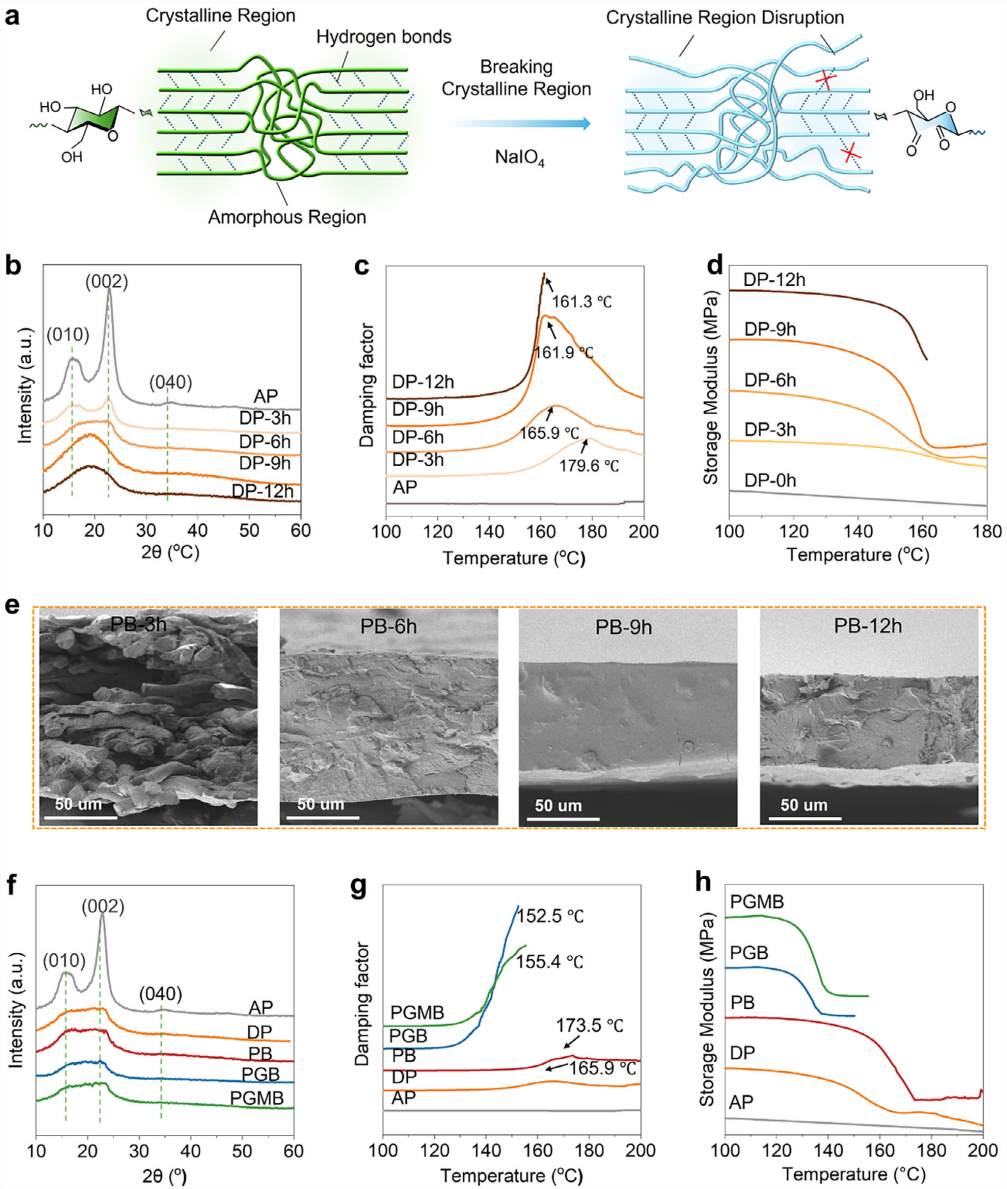
Crytallinity and dynamic analysis of DPs and wastepaper‐based bioplastics. (a) Schematic illustration of the disruption of crystalline domains in virgin cellulose induced by NaIO_4_ oxidation. (b) XRD characterization, (c) damping factors, and (d) storage modulus of DPs. (e) SEM images of PBs. (f) XRD characterization, (g) damping factors, and (h) storage modulus of AP, DP, and wastepaper‐based bioplastics.

As a semi‐crystalline polymer, cellulose exhibits a relatively high glass transition temperature (*T_g_*) of 200°C–250°C. This temperature is close to or even higher than its thermal degradation temperature; hence, cellulose does not turn soft before decomposition. This is attributed to the densely packed crystalline regions composed of well‐aligned cellulose chains and extensive hydrogen bonding, making thermal processing impossible [21]. Therefore, disrupting the crystalline region is essential to achieve thermal processibility (Figure 3a). The diffraction pattern of the AP displays two distinct peaks at 15.75° and 22.87°, and a weaker peak at 34.87°, corresponding to the (010), (002), and (040) planes of the cellulose I crystal structure (Figure 3b) [31]. Upon NaIO_4_ treatment, these peaks in the DPs gradually diminish with increasing oxidation time, as oxidation begins on the crystalline surface and IO_4_^−^ ions progressively penetrate and disrupt the crystalline core [29]. The amorphous region, being less ordered, is more susceptible to oxidation; partial cleavage of glycosidic bonds occurs there first, leading to chain scission and a reduction in molecular weight [32].

Dynamic mechanical analysis (DMA) was conducted to further examine the thermal behavior of DPs. The damping factor (tan δ) curves show clear *T_g_* peaks that decrease from 179.6°C to 161.9°C with increasing oxidation time (Figure 3c). Similarly, the storage modulus (G′) decreases with temperature, with a more pronounced decline observed for longer oxidation durations (Figure 3d). The loss modulus (G′′) exhibits the same downward shift near *T_g_* (Figure S10a). In contrast, no *T_g_* signal is observed for AP below 200°C. These results indicate enhanced molecular mobility in the oxidized cellulose chains, largely due to the partial dissociation of hydrogen bonds upon heating. Longer oxidation times further reduce hydrogen bonding and ring structures, increasing chain flexibility and lowering the energy required for deformation. Notably, the DP‐12h sample fractured at 161.3°C during DMA testing, likely because its high plasticity prevented it from sustaining tensile stress at elevated temperatures. Consequently, hot‐pressed paper‐based bioplastics (PBs) derived from DPs exhibit distinct oxidation‐dependent microstructures. The least oxidized sample (PB‐3h) shows the greatest thickness and roughest surface, with visible fibers and pores (Figure 3e; Figure S11). Increasing oxidation time yields denser and smoother structures as higher oxidation enhances fiber plasticity. These findings underscore the tunable viscoelasticity of DPs, enabling adjustable thermal processibility.

As well as PB, DP can be further transformed into plasticized PGB and hydrophobically modified plasticized bioplastics (PGMB). Compared to PB, the crystalline peaks of PGB and PGMB slightly decrease, suggesting that glycerol addition further disrupts the crystalline regions (Figure 3f). A distinct *T_g_* peak appears in the tan δ curve of PB (Figure 3g), accompanied by a gradual decrease in G′ and visible deformation under tensile loading after DMA testing (Figure 3h; Figure S12a), confirming that hot‐pressed PBs remain thermally processable. However, PB exhibits a *T_g_* of 173.5°C—higher than that of DP (165.9°C)—due to increased intermolecular hydrogen bonding and covalent linkages (e.g., hemiacetal and hemialdal) formed during hot pressing, consistent with NMR results. In contrast, both PGB and PGMB display steeper modulus slopes and lower *T_g_* values (Figure S10b), as glycerol acts as a plasticizer that enhances thermal plasticity, evidenced by their mechanical failure at elevated temperatures (Figure S12b).

### 2.3 Mechanical Performance

**FIGURE 4:**
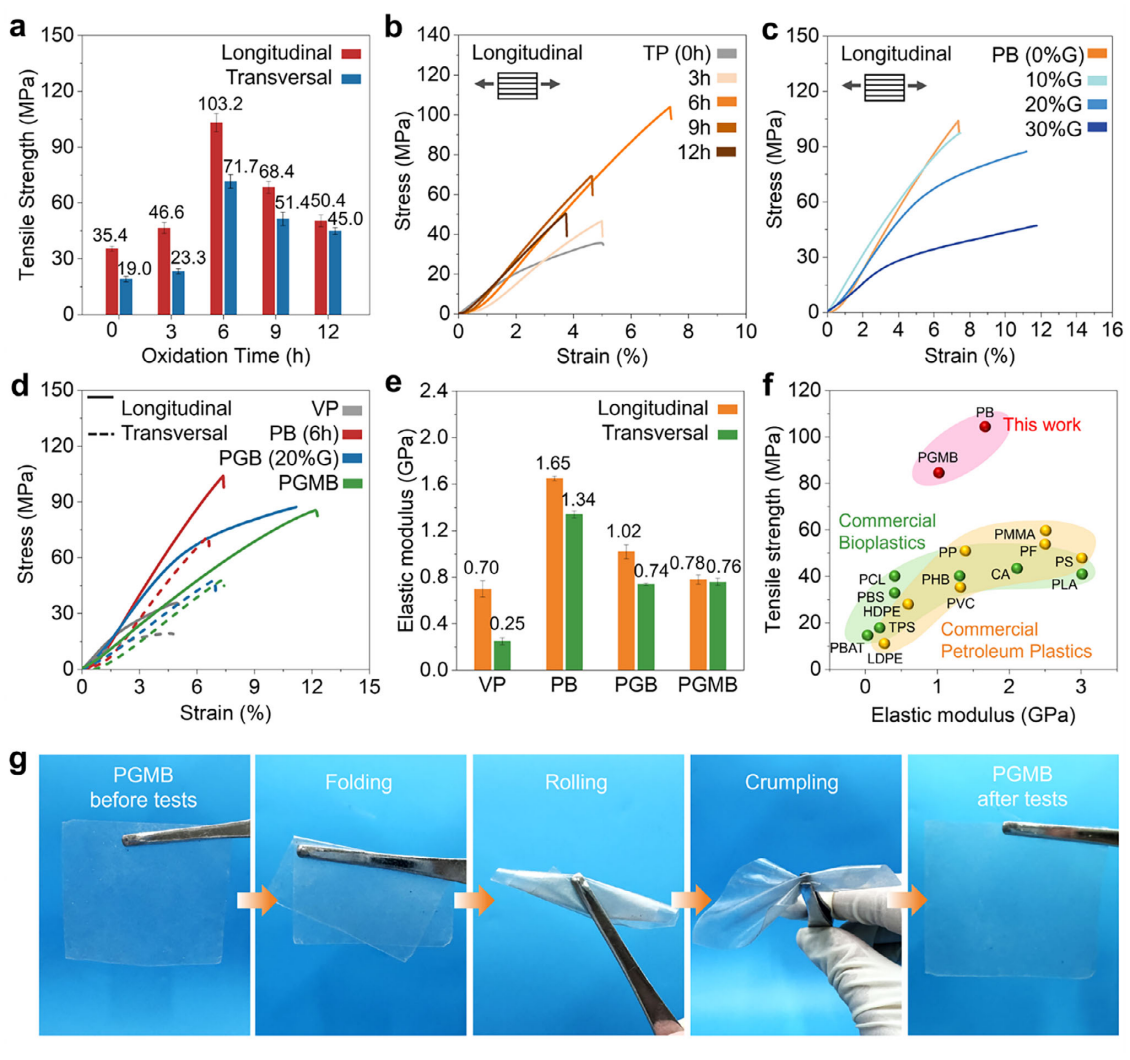
Mechanical properties of wastepaper‐based bioplastics. (a) Tensile strength and (b) stress–strain curves of PBs with various oxidation times. (c) Stress–strain curves of PGBs with different glycerol content. (d) Tensile strength and (e) elastic modulus of VP and wastepaper‐based bioplastics. (f) Comparative analysis of the mechanical performance of PB and PGMB with representative commercial petroleum‐based plastics and bioplastics. (g) Photographs of PGMB before, during, and after folding, rolling, and crumpling tests.

To quantitatively evaluate the effect of oxidation on the mechanical properties of hot‐pressed PBs, tensile tests were conducted in both longitudinal and transverse directions. As oxidation time increased, tensile strength improved, peaking at PB‐6h with values of 103.2 MPa (longitudinal) and 71.7 MPa (transverse)—2.9 and 3.8 times higher than those of VP, respectively (Figure 4a). PB‐6h also exhibited the highest elastic modulus, reaching 1.65 GPa in the longitudinal and 1.34 GPa in the transverse direction (Figure S13a). This enhancement is attributed to improved fiber fusion resulting from disrupted crystalline regions and the formation of abundant internal chemical bonds during oxidation [33]. However, excessive oxidation beyond 6 h caused a decline in strength and modulus, likely due to over‐cleavage of glycosidic bonds [22]. PB‐6h also showed the greatest elongation at break—7.3% (longitudinal) and 6.6% (transverse)—owing to increased chain mobility. Further oxidation reduced flexibility by restricting chain movement through additional crosslinking (Figure 4b; Figure S13b).

With glycerol incorporation, the elongation at break of PGB increased from 7.3% in PB to 7.5%, 11.2%, and 11.8% for PGB‐10%G, PGB‐20%G, and PGB‐30%G, respectively, in the longitudinal direction, with a similar trend in the transverse direction (Figure 4c; Figure S13c). This enhanced flexibility arises from three synergistic effects: (1) glycerol expands intermolecular spacing, increasing free volume; (2) it acts as a molecular lubricant, reducing interchain friction and promoting chain mobility, as evidenced by the pronounced fiber pull‐outs observed on PGB and PGMB fracture surfaces (Figure S14); and (3) it forms hydrogen bonds with cellulose, suppressing rigid covalent crosslink formation. These effects, however, also lead to decreased tensile strength and modulus with higher glycerol content (Figure S13d). Despite this trade‐off, MTMS‐coated PGMB retains excellent mechanical robustness, showing tensile strengths of 85.7 MPa (longitudinal) and 48.4 MPa (transverse), and elastic moduli of 0.78 and 0.76 GPa, respectively (Figure 4d,e). As demonstrated by the EDS mapping and XPS Si 2p spectra, a thin MTMS coating was formed on the surface of PGMB, which further improved its flexibility, resulting in elongations at break of 12.2% and 7.4% in the longitudinal and transverse directions, respectively (Figure S15). Overall, glycerol and MTMS modification provides tunable mechanical properties in wastepaper‐based bioplastics, broadening their applicability across diverse functional requirements.

To further validate the superior mechanical performance of our wastepaper‐derived bioplastics, a comparative analysis with common petroleum‐based plastics and bioplastics is presented in Figure 4f. Both PB and PGMB exhibit tensile strengths far exceeding those of most commercial plastics, along with favorable elastic moduli, highlighting their promise as high‐performance, sustainable alternatives to conventional bioplastics that often suffer from limited mechanical robustness. Moreover, the excellent balance of strength and flexibility allows the bioplastics to withstand repeated folding, rolling, and crumpling without visible cracking or fragmentation (Figure 4g). This remarkable mechanical resilience underscores their potential for a broad range of practical applications—from rigid structural components to flexible packaging materials.

### 2.4 Water∖Organic Solvent Stability

**FIGURE 5:**
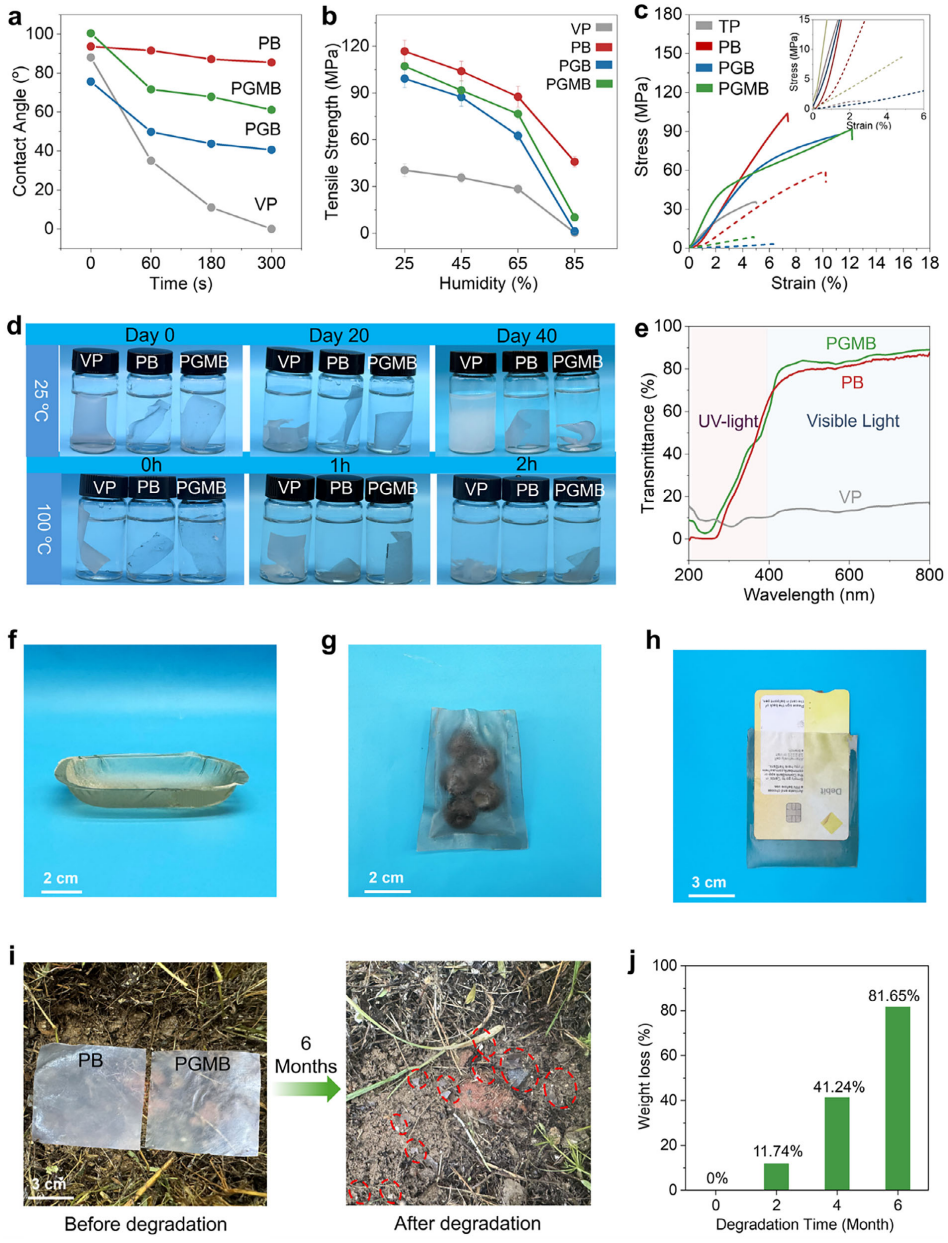
Water resistance, optical transmittance and application of wastepaper‐based bioplastics. (a) Water contact angles of VP and wastepaper‐based bioplastics. (b) Tensile strengths of VP and wastepaper‐based bioplastics under varied RH. (c) Stress–strain curves of VP and wastepaper‐based bioplastics before and after 1 h of water immersion. (d) Photographs of VP, PB, and PGMB after immersion in water at 25°C for 40 days and at 100°C for 2 h. (e) Optical transmittance of VP, PB, and PGMB. Representative applications including (f) a PB container, (g) an adhesive‐free PGMB bag, and (h) a card protective shell fabricated via this strategy. (i) Photographs of PB and PGMB before and after biodegradation. (j) Weight loss of PB and PGMB buried in soil over time.

Water resistance is a key requirement for the practical deployment of bioplastics, yet it remains a major challenge for cellulose‐based materials due to their abundant hydrophilic hydroxyl groups, which limit usability under humid or wet conditions. The as‐developed wastepaper‐based bioplastics, however, exhibit outstanding water resistance. As shown in Figure 5a and Figure S16, PB displays an initial water contact angle (WCA) of 93.6°, remaining as high as 85.4° after 300 s, whereas untreated VP rapidly drops to nearly zero. The incorporation of hydroxyl‐rich glycerol in PGB compromises this hydrophobicity, resulting in reduced water resistance and diminished mechanical stability upon moisture exposure. To address this limitation, PGB was treated with MTMS to enhance surface hydrophobicity. The resulting PGMB exhibits the highest initial WCA and maintains superior hydrophobicity over time, outperforming all untreated counterparts.

The long‐term moisture resistance of the bioplastics was further examined by mechanical testing under different relative humidity (RH) conditions. As RH increased from 25% to 85%, PB retained a tensile strength of ∼45.9 MPa (Figure 5b) and maintained 59.5 MPa—over half its dry‐state strength (103.2 MPa)—even after 1 h of water immersion (Figure 5c). PB also exhibited the lowest water uptake, attributed to the substitution of hydrophilic ─OH groups with ─CHO in dialdehyde cellulose and the formation of a dense, compact structure during hot pressing (Figure S17). Similarly, PGMB demonstrated improved wet strength and reduced water absorption compared with PGB under both high‐humidity and immersion conditions. Collectively, these results indicate that chemical modification, surface hydrophobization, and structural densification act synergistically to impart excellent moisture resistance and environmental durability to the wastepaper‐based bioplastics.

When water droplets were applied to the bioplastic surfaces, droplets readily rolled off PB and PGMB, while VP retained visible water trails, indicating persistent moisture retention (Figure S18). Long‐term immersion tests further confirmed their exceptional water durability: both PB and PGMB remained intact after 40 days in water, with PB largely retaining its rigidity (Figure 5d). Remarkably, both materials also withstood boiling water for 2 h, remaining structurally intact—albeit slightly softened—while VP completely disintegrated. In addition, PB and PGMB exhibited excellent solvent resistance, maintaining their shape and appearance after 60 days of immersion in various organic solvents (Figure S19). These findings collectively demonstrate that the wastepaper‐derived bioplastics possess outstanding environmental stability and moisture durability, positioning them as strong candidates for demanding applications. Furthermore, they exhibit a water vapor transmission rate (WVTR) exceeding 700 g m^−2^ day^−1^ and an oxygen transmission rate (OTR) above 30 cm^3^ m^−2^ day^−1^—superior to many conventional bioplastics such as cellulose films and PBAT (Figure S20) [34, 35, 36]. These barrier properties make them promising for packaging applications with moderate moisture and oxygen barrier requirements, such as cheese, baked goods, and certain fruits and vegetables [37].

### 2.5 Optical Transmittance, Healability, Biodegradability and Environmental Impact Analysis

Compared with VP, both PB and PGMB exhibit markedly higher transparency, allowing underlying patterns to remain clearly visible when covered by the films—demonstrating their potential for transparent packaging applications (Figure S21). To quantitatively assess their optical properties, UV–vis transmittance spectra were recorded (Figure 5e). Both PB and PGMB effectively block UV light in the 200–380 nm range while maintaining high transmittance in the visible region (380–800 nm), indicating that the wastepaper‐based bioplastics combine excellent transparency with UV‐shielding capability. Taking advantage of their outstanding optical clarity and thermal processibility, a variety of packaging prototypes were fabricated from DPs. A rigid PB container was produced by hot pressing, suitable for storing tea leaves and fruits owing to its high mechanical strength and stiffness (Figure 5f; Figure S22). Similarly, a transparent, rigid PB‐based spoon was prepared (Figure S23). Owing to the high ductility and heat‐induced self‐healing ability, a self‐sealed PGMB packaging bag was fabricated without any adhesive (Figure 5g). Furthermore, a transparent, flexible, and self‐sealed PGMB card protector was successfully constructed (Figure 5h).

Notably, this fabrication strategy is not only applicable to intact wastepaper but also effective for damaged or fragmented papers. For instance, a piece of cracked waste print paper was readily converted into a flat, transparent, and intact bioplastic film (Figure S24). Residual printing ink or oil was easily removed by gentle scraping after oxidation, and surface cracks nearly disappeared after hot pressing. Moreover, multiple separated wastepaper fragments could be hot‐pressed into a large, seamless, transparent, and flexible bioplastic film without visible junctions, demonstrating excellent interfacial fusion and surface uniformity (Figure S25). These results highlight the scalability and versatility of our method, enabling efficient upcycling of diverse wastepaper types—including broken, printed, and small‐sized pieces—into high‐performance bioplastics. Additionally, the as‐prepared bioplastics exhibit excellent biodegradability in soil. After six months of burial, the samples degraded into small debris (highlighted by yellow circles in Figure 5i), with a weight loss of 81.65% (Figure 5j; Figure S26). This substantial degradation confirms their environmental sustainability and reinforces their potential as eco‐friendly alternatives to conventional plastic packaging materials.

To further assess the environmental impact of the cellulose‐based bioplastics developed in this work, a cradle‐to‐gate life cycle assessment (LCA) was conducted. The functional unit was defined as 1 kg of final product. The system boundary includes three main steps: pre‐treatment (raw material acquisition and alkali/urea pretreatment), modification (oxidation, plasticization, and surface modification), and fabrication (hot‐pressing) (Figure S27a). Environmental impacts were evaluated in terms of ozone depletion, global warming, freshwater eutrophication, and marine eutrophication (Figure S27b). Conventional petroleum‐based plastics (PET, PP) and bio‐based plastic (PLA) were selected for comparison. Our cellulose‐based bioplastics (PB and PGMB) exhibit significantly lower environmental impacts than conventional petroleum‐based plastics (PET and PP) and even outperform PLA in most categories. These results demonstrate that the developed cellulose‐based materials offer clear environmental benefits, particularly in reducing greenhouse gas emissions and nutrient‐related environmental impacts.

## 3 Conclusion

We have developed thermally processable, transparent, and high‐performance bioplastics directly from wastepaper via a straightforward hot‐press transformation strategy. By converting glucose rings into linear chains and substituting hydroxyl groups with aldehydes, the modified cellulose in wastepaper exhibits enhanced molecular mobility, resulting in a lower *T_g_* and excellent thermal processability. This enables the direct hot‐pressing of wastepaper into bioplastics with high optical transparency, strong self‐sealing ability, and scalable fabrication. The resulting well‐fused structure delivers superior mechanical properties, surpassing those of most commercial plastics. Incorporation of glycerol and MTMS further enhances ductility and flexibility, making the bioplastic material suitable for adhesive‐free, heat‐sealed packaging. Crucially, the bioplastics show great biodegradability under natural conditions, demonstrating their minimal environmental impact. This direct conversion strategy provides a scalable route to transform wastepaper into sustainable, high‐performance bioplastics, offering a promising alternative to petroleum‐based plastics and current bioplastics with inherent limitations.

## 4 Experimental Section

### 4.1 Materials and Chemicals

Wastepaper was purchased from Winc Australia Pty. Urea, sodium hydroxide (NaOH), and ethanol (99%) were purchased from Shanghai Aladdin Biochemical Technology Co., Ltd. Citric acid, sodium periodate (NaIO_4_, 99.5%), Glycerol, and methyltrimethoxysilane (MTMS) were purchased from Shanghai Macklin Biochemical Co., Ltd.

### 4.2 Preparation of AP

A 500 mL mixed solution of 7% NaOH and 12% urea was prepared by dissolving NaOH (35 g) and urea (60 g) in deionized water (405 g). The solution was then cooled to −15°C. Wastepaper was immersed in the chilled solution for 30 s, then immediately transferred to a 5% citric acid solution. After soaking in the citric acid solution for 30 min, the paper was thoroughly washed with deionized water several times to remove residual chemicals, yielding the alkali‐treated paper.

### 4.3 Preparation of DP and PB

Alkali‐treated paper was oxidized by immersing it in a 2 wt.% sodium periodate (NaIO_4_) solution (500 mL) and reacting at 50°C in the dark to prevent photodegradation. Oxidation was conducted for 3, 6, 9, and 12 h to obtain DPs with varying degrees of oxidation, designated as DP‐*x*h (where *x* indicates the oxidation duration in hours). After being slightly air‐dried, the oxidized papers were hot‐pressed at 110°C under a pressure of 0.8 MPa for 15 min to produce corresponding paper‐based bioplastics (PB‐xh). Among these, PB‐6h exhibited optimal mechanical performance based on tensile testing and was therefore selected, along with its precursor DP‐6h, for detailed characterization and further modification.

### 4.4 Preparation of PGB and PGMB

Glycerol solutions with concentrations of 10, 20, and 30 wt.% were prepared at room temperature. DPs were individually immersed in these solutions for 15 min to incorporate varying amounts of glycerol. The glycerol‐treated papers were then hot‐pressed at 110°C under 0.8 MPa for 15 min to form paper/glycerol‐based bioplastics, designated as PGB‐*x*%, where *x* refers to the glycerol concentration. Among these, PGB‐20% exhibited an optimal balance between mechanical strength and flexibility and was therefore selected for further modification. For hydrophobic enhancement, a methyltrimethoxysilane (MTMS) solution was prepared at a volume ratio of MTMS:ethanol:deionized water = 1:8:1. The PGB‐20% sample was immersed in the MTMS solution for 10 s and subsequently air‐dried, yielding a modified paper/glycerol/MTMS‐based bioplastic, referred to as PGMB.

### 4.5 Characterizations

To investigate the chemical structure of VP, DPs, and all wastepaper‐derived bioplastics (PB, PGB, and PGMB), Fourier‐transform infrared (FTIR) spectroscopy was conducted using a Nicolet iS50 spectrometer (Thermo Fisher Scientific, USA) in the range of 400–4000 cm^−1^. Solid‐state ^13^C nuclear magnetic resonance (NMR) spectroscopy (AVANCE NEO 600M, Bruker, Germany) was further employed to analyze carbon‐specific structural changes. X‐ray photoelectron spectroscopy (XPS) measurements were performed using an AVANCE NEO 400 (Bruker, Germany) to evaluate elemental composition and chemical bonding variations. The aldehyde content in DPs was quantified via titration, following a previously reported method [33].

Surface morphology and microstructure were examined using scanning electron microscopy (SEM, SU8010, Hitachi, Japan) after sputter‐coating the samples with gold. The crystalline structure was characterized by X‐ray diffraction (XRD, XRD‐6000, Shimadzu, Japan) with a scanning range of 10°–60° and a scanning speed of 5°/min. Thermal properties were assessed using differential scanning calorimetry (DSC, Q200, TA Instruments, USA) at a heating rate of 10°C/min. Dynamic mechanical analysis (DMA, Q800, TA Instruments, USA) was carried out under tensile mode to evaluate temperature‐dependent mechanical behavior, with a heating rate of 2°C/min. Water resistance was assessed by measuring water contact angles (WCA) using a contact angle meter (JY‐82, Hake, China). Optical transparency was evaluated with an UV–vis–NIR spectrophotometer (LAMBDA 950, PerkinElmer, USA) across the 200–800 nm wavelength range. The oxygen permeability of the bioplastics was measured using a gas permeability tester via the differential pressure method in accordance with GB/T 1038. Water vapor transmission rate (WVTR) was determined using a water vapor permeability tester following the FB/T 1037 standard.

### 4.6 Mechanical Tensile Test

Specimens with a width of 3 mm were prepared for tensile testing, and their thickness was determined via SEM imaging. For each sample type, 3–5 replicates were tested to ensure reproducibility. To evaluate the mechanical performance of the bioplastics under different moisture conditions, samples were preconditioned at relative humidity (RH) levels of 25%, 45%, 65%, and 85% for 24 h prior to testing. To assess the effect of water immersion, additional samples were soaked in deionized water for 1 h before testing. Tensile tests were performed using an Instron 5569 universal testing machine with a crosshead speed of 2 mm/min and a gauge length (span) of 5 mm.

### 4.7 Water Absorption Test

The water absorption capacity of the bioplastic samples was evaluated by measuring their mass before and after immersion in water for 48 h. The samples were gently wiped to remove surface water before the final mass measurement. Water absorption was calculated using the following equation:

$$w\;\left(\%\right)=\frac{M_{1}-M_{0}}{M_{0}}\times 100\%$$

where *w* is the water absorption, *M_0_* is the initial dry mass, and *M_1_* is the mass after water absorption.

## Author Contributions

P.S. and Z.Z. conceived and designed the study, carried out the material synthesis, characterizations and writing. All authors contribute to the manuscript revision.

## Conflicts of Interest

The authors declare that they have no competing interests.

## Supporting information




**Supporting File**: advs75978‐sup‐0001‐SuppMat.docx.

## Data Availability

Data sharing not applicable to this article as no datasets were generated or analysed during the current study.
