# Supplementary material for: Thermally Processable, Transparent, Mechanically Tunable and Robust Bioplastics From Wastepaper
Source: Adv Sci (Weinh). 2026 Jun 9;13(49):e75978. doi: 10.1002/advs.75978 (PMC13336872; doi:10.1002/advs.75978)
Supplement: Supplementary file 1 [file ADVS-13-e75978-s001.docx]

Supporting Information

**Thermally Processable, Transparent, Mechanically Tunable and Robust Bioplastics from Wastepaper**

*Zhezhe Zhou, Tao Chu, Boyou Hou, Mark Lynch, Siqi Huo, Min Hong, Jianguo Yang, Polly Burey,* *Qiang Gao***, Guobo Huang, Pingan Song**

**Contents**

**Figures S1 - S26**

**Figure S1**. Photographs and SEM images of a) VP and b) alkali-treated paper.

**Figure S2**. a) Specific surface area, b) XRD characterization and c) weight of VP and AP.

**Figure S3**. a) Schematic diagram of anisotropic VP; b) tensile strength of VP and AP in two directions; surface SEM images of (c) VP and (d) AP after hot pressing.

**Figure S4**. Photographs of VP after NaIO_4_ oxidation.

**Figure S5**. Photographs of DPs with various oxidation hours.

**Figure S6**. SEM images of DPs with various oxidation hours.

**Figure S7**. The aldehyde content of DPs with various oxidation hours.

**Figure S8**. The chemical structures of cellulose dialdehyde, hydrated cellulose aldehyde, cellulose hemialdal and cellulose hemiacetal.

**Figure S9**. XPS spectra of a) PGB and b) PGMB.

**Figure S10**. Loss modulus of a) DPs and b) wastepaper-based bioplastics.

**Figure S11**. SEM images of the surfaces of PBs converted from DPs with various oxidation times.

**Figure S12**. Photographs of a) PB and b) PGMB after DMA test.

**Figure S13**. a) Elastic modulus and b) stress–strain curves of PBs; c) stress–strain curves in the transverse direction and d) elastic modulus of PGMBs with varying glycerol contents.

**Figure S14**. The cross-section SEM images of a) PB, b) PGB and c) PGMB.

**Figure S15**. a) Cross-sectional SEM image, b) EDS image, and c) Si Kα elemental mapping of PGMB. d) XPS spectra of PGMB.

**Figure S16.** The water contact angles of wastepaper-based bioplastics.

**Figure S17**. Water absorption of VP and wastepaper-based bioplastics.

**Figure S18**. Photographs of the surface of VP, PB and PGMB before and after water dripping.

**Figure S19**. Organic solvent resistance of PB and PGMB.

**Figure S20**. Oxygen transmission rate (OTR) and water vapor transmission rate (WVTR) of PB and PGMB.

**Figure S21**. Photographs of VP, PB and PGMB covering on the colorful pattern.

**Figure S22**. Photographs of the PB container.

**Figure S23**. Photograph of the PB spoon.

**Figure S24**. Process of converting broken wastepaper into an intact bioplastic.

**Figure S25**. a) Process of assembling separate DPs into an intact bioplastic by simple hot pressing. b) Transparency, integrity, and flexibility of the assembled bioplastic.

**Figure S26**. Photograph of the bioplastic debris after six months of degradation.

**Figure S27**. Life cycle assessment. a) System boundary of PB and PGMB preparation process. b) Environmental impacts of 1 kg PET, PP, PLA, PB and PGMB.
